# Business as Usual: A Lack of Institutional Innovation in Global Health Governance: Comment on "Global Health Governance Challenges 2016 – Are We Ready?"

**DOI:** 10.15171/ijhpm.2016.113

**Published:** 2016-08-17

**Authors:** Kelley Lee

**Affiliations:** Faculty of Health Sciences, Simon Fraser University, Burnaby, BC, Canada.

**Keywords:** Global Health Governance (GHG), World Health Organization (WHO) Reform, Globalization, Institutional Innovation

## Abstract

There were once again high expectations that a major global health event - the Ebola virus outbreak of 2014-2015 - would trigger meaningfully World Health Organization (WHO) reform and strengthen global health governance (GHG). Rather than a "turning point," however, the global community has gone back to business as usual. This has occurred against a backdrop of worldwide political turmoil, characterised by a growing rejection of existing political leaders and state-centric institutions. Debates about GHG so far have given insufficient attention to the need for institutional innovation. This entails rethinking the traditional bureaucratic model of postwar intergovernmental organizations which is disconnected from the transboundary, fast-paced nature of today’s globalizing world.

More than halfway through 2016, a year Ilona Kickbusch describes as “a turning point for global health” amid “new political realities and global insecurities,”^1^ there are indeed signs of change. However, the direction of travels appears to be away from collective action. Right- and left-wing populist movements across many western liberal democracies (spanning the Syriza Party in Greece to the National Front in France) have expressed their voices, for example, through the UK referendum vote to leave the European Union (EU), and nomination of Donald Trump as the Republican Party’s US presidential candidate. Many see these developments as a populist rejection of globalization.^2,3^ Meanwhile, the issues requiring global cooperation continue unabated including major conflicts across the Middle East; unprecedented migration flows; volatility of global financial markets; and a perilously warming world (July 2016 being the hottest on record).

Given this crowded global agenda, the Ebola debacle has faded from recent memory and global health has retreated from the world’s headlines. Attention has shifted elsewhere and it is difficult to spot a “turning point,” or even the blinking of a turn signal, in global health governance (GHG). There are a few who remain hopeful for meaningful reform of the World Health Organization (WHO) signalled, for example, by the creation of the Health Emergencies Programme “to help countries prepare for, prevent, respond to and recover from emergencies quickly, in a more predictable, dependable and accountable way, whether these are caused by disease outbreaks, disasters or conflict.”^4^ There are many others who are sceptical that the organization has the capacity to reform itself.^5-7^ WHO’s formal mandate and structure remains fundamentally unchanged. Member states refuse to increase resources or adopt a different funding model. Painfully familiar conversations have ensued or, worse, gone silent.

## Business as Usual: An Outmoded State

Kickbusch is correct that many of the multilateral institutions created after the Second World War, to maintain international peace and security, are obsolete. The United Nations (UN) system, along with the thousands of intergovernmental (or international governmental) organizations created since 1945, are an extension of the European notion of the “state” as it evolved out of the Peace of Westphalia (1648). As Richard Falk writes:

“*One of the seemingly permanent contributions of Europe to the manner of organizing international society was to create a strong consensus in support of the idea that only a territorially delimited sovereign state is entitled to the full privileges of membership. The United Nations [UN], the institutional embodiment of international society, recognizes this principle by limiting membership in the Organization to ‘states*.’”^8^

This state-based notion of world order remains embedded in international law and organizational theory, as well as, efforts to understand global insecurity and strengthen collective action.

In this context, politics continues to be primarily defined in terms of states and their relative power. Kickbusch refers to a “power shift” towards emerging economies, and a reconfiguration of the international order – from postwar superpower rivalry, to the United States as “lonely superpower,”^9^ to a multipolar world.^1^ On the one hand, multipolarity is expected to generate a degree of friction as states jostle and settle into new positions of political and economic power. Consensus can be more difficult to achieve in international negotiations while this shifting occurs. On the other hand, Kickbusch describes a “diffusion” of power to more states, and with it, new opportunities for collective action. While traditional aid donors may retreat to prioritising “national interests and foreign policy goals,” increased South-South cooperation offers new potential. She concludes that “a much wider range of countries now possesses the means that are constitutive for participation in global governance: endogenous resources, transnational connectivity, and geopolitical status.”^1^ The crowded calendar of intergovernmental meetings to address climate change, trade and investment, sustainable development, transnational crime, corporate accountability, and umpteen other global issues seems to provide evidence of the robust health of global governance.

Winston Churchill famously stated that it is always better to “jaw, jaw than to war, war.”^10^ Good governance, however, needs to be measured by more than the number of meetings where jawing occurs, but also who’s doing the talking. The problem with casting global problems in terms of the divergent interests, unequal resources and resultant conflicts of states, and their solutions as primarily dependent on states negotiating with other states, is the glaring neglect of interests, ideas and institutions that do not conform to national borders. International relations in the early 21st century is more fraught, not just because some states are getting stronger, and others are getting weaker, but because political identity and interests are more complex. And Westphalian-based political institutions alone are unable to represent and mediate among them. In other words, understanding the “new political realities” emerging must go beyond scorecards of which states are rising and falling, the growth of newly emerging economies, and the shift to multipolarity. If we are to reframe debates about GHG, we need to recognise the disconnect between our political institutions and new polities emerging. We cannot continue business as usual but need to recognise that there are deeper political changes afoot.

## Global Change in an Era of Angry Politics

The real “political realities” challenging global governance stem from the acceleration of neoliberal-based market globalization, since the late twentieth century, which has restructured the global political economy in ways suited to the interests of the few over the many. Unprecedented wealth has been generated, but the ensuing costs and benefits are being unfairly distributed. The first concerted push back against this new world order arose during the 1999 World Trade Organization (WTO) Ministerial Conference which surprised negotiators by its ferocity and political breadth. Negotiations were eventually relaunched as the Doha Development Agenda, with a continued belief that “global problems can be addressed through a multilateral framework” of WTO’s then 142 member states.^11^ In 2011, the Occupy Movement formed to oppose a global financial system dominated by large corporations, and characterised by inequality and instability. The claims that a multipolar world is emerging positively imply a wider sharing of power among a greater number of states. However, this is belied by the fact that the wealth of 62 people is equivalent to that owned by half the world’s population.^12^ Intergovernmental governance is seen as failing to redress, and indeed forms part of, the structural conditions which facilitate stark inequities.^13^ While the Occupy Movement was criticised for lacking vision and depth, and soon dispersed without achieving real change, political disenchantment has continued to simmer among “the 99%” buffeted by globalization. The leak of 11.5 million offshore financial records in April 2016, known as the Panama Papers, seems to confirm that systemic flaws in global capitalism indeed stack the cards in favour of the super rich and powerful.^14^

In 2016, therefore, the “turning point” reached may be that sufficient numbers of the voting public across the political spectrum are so unhappy with their political leaders and institutions that they are seeking radical change. On the political left, many have lost faith in the capacity of existing political institutions to serve the public interest and ensure social equity. On the political right, governments are seen as failing to protect local jobs, control immigration, and thus, national interests. As one article in *Der Spiegel* summed up: *“Whether they are fans of Donald Trump, supporters of Brexit or Marine Le Pen voters in France, ‘angry voters’ have one thing in common: They have been left in the dust by globalization.”*^15^

## Implications for Global Health Governance: The Innovation Gap

The search for effective GHG cannot be separated from this worldwide political turmoil. When WHO was established in 1948, its design and agreed functions reflected a desire to re-establish and maintain a peaceful world order built on an international states system. Governed by the principle of one state, one vote, the political bodies of WHO (namely the World Health Assembly, Executive Board and regional committees) are intended to provide member states the opportunity to collectively set goals, agree priorities and allocate resources. The postwar design of WHO in 1948 also adopted Max Weber’s bureaucratic model which consists of a functional hierarchy of authority, a departmental separation of duties, standardised procedures, and an established set of policies or rules.^16^

Fast forward almost 70 years and the political limitations of WHO’s postwar structure are woefully apparent. The organization might point to its 193 member states and claim to be universally representative, but it is far from politically inclusive. Like the political alienation felt by millions around the world, many members of the global health community has turned elsewhere to move issues forward and get things done. Moreover, WHO’s Weberian bureaucracy worked well during periods of stability or slow change. However, as Jamali et al write: “weaknesses were gradually exposed with accelerating globalization and technological innovation.”^17^

In the business world, successful firms adapt to change through institutional innovation in the form of new organizational models and management paradigms. Institutional innovation concerns “redefining the rationale for institutions and developing new relationship architectures within and across institutions to break existing performance trade-offs and expand the realm of what is possible….[thus] creating smarter institutions that can thrive in a world of exponential change”.^18^ On the occasion of its one-hundredth birthday, IBM ran an advertisement in the *New York Times* which read:

“*Nearly all the companies our grandparents admired have disappeared. Of the top 25 industrial corporations in the United States in 1900, only two remained on that list at the start of the 1960s. And of the top 25 companies on the fortune 500 in 1961, only six remain there today*….”^19^

In fact, the situation is even grimmer. As Denning writes, the average life expectancy of a Fortune 500 company has declined from around 75 years half a century ago, to less than 15 years today. The reason is the accelerated pace of uncertainty and change in the world economy.^20^ The clear lesson for firms has been to adapt or die.

What we see is a steady decline of WHO, clinging furiously to obsolete political institutions and bureaucratic model, yet kept alive by member states as an essential public institution. This decline is not because WHO is not needed, but because it has not adapted to a changing world. It is not the WHO that we need today. In other parts of the health sector, innovation thinking has been embraced. We see an explosion of novel applications of new technologies for healthcare or healthcare systems. Mobile devices, for example, are being used for everything from diagnostics, to healthy lifestyle messaging, to post operative monitoring. We also see innovation thinking in the planetary conceptualisation of health problems and their solutions. A good example is the Blue Dot project at the University of Toronto, Toronto, ON, Canada which brings together geographic information systems, spatial analytics, data visualization, and computer science to model how infectious diseases spread and impact populations worldwide.^21^ Beyond the health sector, the world is swirling with institutional innovations in this “age of post-bureaucracy”^16^– crowd funding, open source learning, and the sharing economy are a few examples. An increasing number of examples come from the public sector.^22^ Political innovation must become a fundamental part of this conversation. How might open source learning be used to strengthen the capacity to participate in global health politics? How might virtual and interactive town halls improve communication between global health policy-makers and the constituencies they serve? How might the closed world of global policy-making be opened up and strengthened through virtual public consultations, feedback systems and monitoring systems? How might the concept of global citizenship become institutionalized within our global health institutions?

## Conclusion

Debates about WHO reform and strengthening GHG to date have been insufficiently informed by institutional innovation thinking. Both diagnoses and design of remedial measures continue to focus on the state and intergovernmental institutions. This approach, limited to tinkering with a bureaucratic model inherited from the postwar era, seems strangely detached from the broader political turmoil unfolding around the world. Globalization has created new collective health needs which cross old spatial, temporal and cognitive boundaries. In response, we need GHG institutions which represent the many, not the few; are sufficiently nimble to act effectively in a fast-paced world; and capable of bringing together the best ideas and boundary spanning knowledge available.

## Acknowledgements

KL is supported by the National Cancer Institute, US National Institutes of Health, Bethesda, MD, USA (Grant No. R01-CA091021). The author is solely responsible for the contents of this paper.

## Ethical issues

Not applicable.

## Competing interests

Author declares that she has no competing interests.

## Author’s contribution

KL is the single author of the paper.

## References

[1] Kickbusch I (2016). Global health governance challenges 2016 - are we ready? Int J Health Policy Manag.

[2] Elliot L. Brexit is a rejection of globalization. Guardian. June 26, 2016. https://www.theguardian.com/business/2016/jun/26/brexit-is-the-rejection-of-globalisation.

[3] Colvin G. Donald Trump Plays Off Globalization Anger. Fortune. July 22, 2016. http://fortune.com/2016/07/22/donald-trump-plays-off-globalization-anger/.

[4] World Health Organization (WHO). Renewed partnerships build momentum for WHO’s new Health Emergencies Programme. Geneva: WHO; 2016. http://www.who.int/about/who_reform/emergency-capacities/health-emergencies-programme/en/.

[5] Kamradt-Scott A (2016). WHO’s to blame? The World Health Organization and the 2014 Ebola outbreak in West Africa. Third World Q.

[6] Huang YZ. How to Reform the Ailing World Health Organization. Council on Foreign Relations. May 3, 2016. http://www.cfr.org/health/reform-ailing-world-health-organization/p37831.

[7] Garrett L. Secret vote on WHO bodes ill for future of global health. Humanospere. May 23, 2016. http://www.humanosphere.org/world-politics/2016/05/secret-vote-on-who-bodes-ill-for-future-of-global-health/.

[8] Falk R. A New World Order? ISIS and the Sykes-Picot Backlash. Foreign Policy Journal. December 26, 2015. http://www.foreignpolicyjournal.com/2015/12/26/a-new-world-order-isis-and-the-sykes-picot-backlash/.

[9] Huntingdon S (1999). The Lonely Superpower. Foreign Aff.

[10] Churchill W. Remarks at US White House Luncheon, June 26, 1954.

[11] World Trade Organization (WTO). The Doha Development Agenda, Doha launches negotiations, TNC oversees them. Geneva: WTO; 2003. https://www.wto.org/english/thewto_e/minist_e/min03_e/brief_e/brief02_e.htm.

[12] Oxfam. An Economy for the 1%. Oxfam Briefing Paper. January 18, 2016. https://www.oxfam.org/sites/www.oxfam.org/files/file_attachments/bp210-economy-one-percent-tax-havens-180116-en_0.pdf.

[13] Gill S, Benatar S (2016). Global Health Governance and Global Power: A Critical Commentary on the Lancet-University of Oslo Commission Report. Int J Health Serv.

[14] International Consortium of Investigative Journalists. Giant Leak of Offshore Financial Records Exposes Global Array of Crime and Corruption. https://panamapapers.icij.org/20160403-panama-papers-global-overview.html. Published April 2016.

[15] Electorate Tremors: The Era of the Angry Voter Is Upon Us. Der Spiegel. July 6, 2016. http://www.spiegel.de/international/europe/brexit-the-era-of-the-angry-voter-is-upon-us-a-1101438.html.

[16] Weber M. Economy and Society (Reprint). New York: Bedminster Press; 1968.

[17] Jamali D, Khoury G, Sahyoun H (2006). From bureaucratic organizations to learning organizations. The Learning Organization.

[18] Hagel J, Brown J. Institutional Innovation. Deloitte University Press; 2013. http://dupress.com/articles/institutional-innovation/.

[19] IBM. New York Times. June 15, 2011.

[20] Denning S. Why did IBM Survive? Forbes. July 10, 2011. http://www.forbes.com/sites/stevedenning/2011/07/10/why-did-ibm-survive/#292c643719b1.

[21] About BlueDot. BlueDot website. http://bluedot.global/about.

[22] Doner R, ed. Explaining Institutional Innovation, Case Studies from Latin America and East Asia. Social Science Research Council; 2010.

